# RNA Interference Applied to Crustacean Aquaculture

**DOI:** 10.3390/biom14111358

**Published:** 2024-10-25

**Authors:** Carlos Fajardo, Marcos De Donato, Marta Macedo, Patai Charoonnart, Vanvimon Saksmerprome, Luyao Yang, Saul Purton, Juan Miguel Mancera, Benjamin Costas

**Affiliations:** 1 Department of Biology, Faculty of Marine and Environmental Sciences, Instituto Universitario de Investigación Marina (INMAR), Campus de Excelencia Internacional del Mar (CEI-MAR), University of Cadiz (UCA), 11510 Puerto Real, Spain; juanmiguel.mancera@uca.es; 2 Interdisciplinary Centre of Marine and Environmental Research, The University of Porto (CIIMAR), 4450-208 Matosinhos, Portugal; up202202115@edu.icbas.up.pt (M.M.); bcostas@ciimar.up.pt (B.C.); 3 Center for Aquaculture Technologies (CAT), San Diego, CA 92121, USA; mdedonato@aquatechcenter.com; 4 Escuela de Medicina y Ciencias de la Salud, Tecnológico de Monterrey, Querétaro 76130, Mexico; 5 Institute of Biomedical Sciences Abel Salazar (ICBAS), University of Porto (UP), 4050-313 Porto, Portugal; 6 Center of Excellence for Shrimp Molecular Biology and Biotechnology (Centex Shrimp), Faculty of Science, Mahidol University, Bangkok 10400, Thailand; patai.cha@biotec.or.th (P.C.); vanvimon.sak@biotec.or.th (V.S.); 7 National Center for Genetic Engineering and Biotechnology (BIOTEC), National Science and Technology Development Agency (NSTDA), Bangkok 12120, Thailand; 8 Department of Structural and Molecular Biology, University College London (UCL), London WC1E 6BT, UK; luyao.yang.20@ucl.ac.uk (L.Y.); s.purton@ucl.ac.uk (S.P.)

**Keywords:** anti-viral, dsRNA, gene-silencing, immunology, innate immunity, miRNA, nanoencapsulation, RNAi, shrimp, siRNA

## Abstract

RNA interference (RNAi) is a powerful tool that can be used to specifically knock-down gene expression using double-stranded RNA (dsRNA) effector molecules. This approach can be used in aquaculture as an investigation instrument and to improve the immune responses against viral pathogens, among other applications. Although this method was first described in shrimp in the mid-2000s, at present, no practical approach has been developed for the use of dsRNA in shrimp farms, as the limiting factor for farm-scale usage in the aquaculture sector is the lack of cost-effective and simple dsRNA synthesis and administration procedures. Despite these limitations, different RNAi-based approaches have been successfully tested at the laboratory level, with a particular focus on shrimp. The use of RNAi technology is particularly attractive for the shrimp industry because crustaceans do not have an adaptive immune system, making traditional vaccination methods unfeasible. This review summarizes recent studies and the state-of-the-art on the mechanism of action, design, use, and administration methods of dsRNA, as applied to shrimp. In addition, potential constraints that may hinder the deployment of RNAi-based methods in the crustacean aquaculture sector are considered.

## 1. Introduction

The aquaculture sector has rapidly evolved to become not only an important economic activity but also the fastest-growing food production system worldwide [1]. However, due to the intensification of farming conditions, this industry has been heavily affected by a plethora of transboundary aquatic animal pathologies caused by numerous etiological agents, with viruses causing particularly devastating problems. This situation has become even more complex due to the frequent identification of new pathogens [2,3]. Therefore, disease outbreaks have emerged as one of the key challenges for the integral development of this industry around the world [4,5].

The capacity to manage these viral pathogens is currently focused predominantly on pathogen exclusion using specific pathogen-free (SPF) animals and biosecurity procedures [6], as well as the development of stocks by selective breeding programs to generate specific pathogen-resistant (SPR) animals [7]. However, these approaches often have limited success depending on the type of pathogen. Thus, to satisfy the increasing demands of an expanding human population, newer approaches and strategies for disease management need to be developed and deployed [8]. Currently, more practical anti-viral management procedures are not available, mainly because of the lack of an adaptive immune response system in crustaceans, which makes traditional vaccination approaches unsuitable. However, the lack of an acquired immune response means that the innate immunity system is generally highly developed in shrimp and other crustaceans. Comprising diverse cell types and related mechanisms, the innate immune response can cope with pathogens in a non-specific mode, but fails to produce a long-lasting protective immune effect, and instead provides a faster anti-infection response [9].

In this context, one technology that is likely to play an important role in the future development of the aquaculture industry is RNAi. The first description of the RNAi phenomenon dates back to experiments aimed at improving the hue of purple petunias, in which the transgenic delivery of a second copy of the endogenous gene responsible for pigment synthesis produced completely white or mottled flowers, instead of the expected deep-purple color [10]. Subsequently, the same phenomenon was observed when researchers tried to enhance the generation of the orange pigment in the fungus *Neurospora crassa* [11]. In the case of plants, this peculiar effect was initially named co-suppression [10], while in fungi it was called quelling [11].

The first description of RNAi in animals arose by accident when Guo and Kemphues [12] injected antisense strands of RNA into the nematode species *Caenorhabditis elegans* to inhibit *par-1* gene expression. However, the relation between double-stranded RNA (dsRNA) and knock-down of gene expression was first described by Fire et al. [13], who discovered that dsRNA, instead of sense or antisense single-stranded RNA (ssRNA), triggered gene knock-down when microinjected into *C. elegans.* Thenceforth, RNAi has been described as a highly conserved mechanism that is present not only in fungi and protozoa, but also in the more complex life forms of animals and plants [14,15,16,17,18].

RNAi is considered a transcriptional or post-transcriptional gene knock-down mechanism, in which dsRNA corresponding to a transcribed region of a particular gene is delivered into an organism, leading to the elimination of the respective mRNA and, therefore, silencing the expression of that gene [19]. The basic notion of RNAi is based on the degradation of mRNA triggered by the recognition of homologous sequence fragments from the dsRNA, and in the translational suppression by the complementary binding between small RNAs (sRNAs) and the 3′ untranslated region (UTR) of the specific target mRNA [20].

Due to this sequence-specific capacity to lower the expression of specific genes, RNAi has been widely employed to study the function of target genes by silencing their expression without changing the genotype [21]. Moreover, the knock-down capacities could be helpful not only to investigate the function of genes but also to recognize vaccine candidates and drug targets [22], as well as to manage infectious diseases through hindering pathogen proliferation, development, and transmission inside the host [5,21]. Therefore, gene silencing represents a very promising method for the development of therapeutic and/or prophylactic procedures for organisms that have a systemic RNAi machinery, including shrimp [5,23]. At present, direct evidence of siRNA signal amplification in crustaceans remains limited, but there are reports suggesting that the RNAi mechanism in crustaceans might involve signal recycling, which could explain the apparent efficiency of the RNAi response despite the variability in dsRNA dosing. For instance, a study by Yodmuang et al. [24] demonstrated that small amounts of dsRNA could induce long-lasting anti-viral effects in shrimp, hinting at possible amplification or efficient reuse of RNAi components, though not definitively proving a canonical amplification pathway similar to that found in nematodes. Additionally, studies by Attasart et al. [25] and Tirasophon et al. [26] discussed the prolonged effect of dsRNA in shrimp, potentially supporting an amplification or recycling mechanism, though more research is needed to fully elucidate this process. Considering the growing attention that the RNAi mechanism has acquired, this review focuses on presenting the state-of-the-art in relation to this important molecular tool as a potential method for the control of the main pathogens that affect the crustacean-farming sector.

## 2. RNAi Mechanism of Action

The knock-down of gene expression in animals is possible through dsRNA administration by injection, immersion, or feeding [27,28]. Following such treatment, the dsRNA is translocated from the extracellular matrix into the cytoplasm of cells either through the systemic interference defective-1 (SID-1) channel, which is a passive channel specific to dsRNA [29,30,31], or by endocytosis [32].

**Figure 1:**
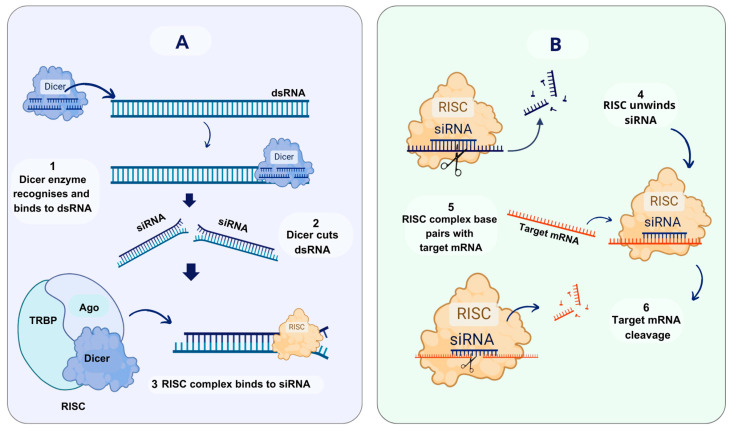
Simplified RNAi mechanism. (**A**) Initiation step and (**B**) effector step.

Biochemical and genetic studies point to a two-step process for RNAi: an initiation step and an effector step [19] (Figure 1). The first step occurs in the cytoplasm, where long dsRNAs (>100 bp), generated from viral, transgenic, or endogenous transcripts, are cleaved into small fragments (21–22 bp), called small-interfering RNA (siRNA). This RNA processing is carried out by Dicer2, a multidomain ribonuclease-III-related enzyme (RNase III) that has the capacity to identify and cut dsRNA at specific sequences or positions [33,34,35]. These siRNA fragments have a peculiar 3′ overhang of two nucleotides (nt) that enables them to be identified by the RNAi enzymatic system, and this subsequently triggers the homology-dependent elimination of the specific target mRNA [36,37,38]. During the second step, siRNAs are integrated into a multi-protein RNA-induced silencing complex (RISC) by the action of the protein Argonaute (Ago), which is the key element of RISC. Subsequently, and in its unwound state, the sense strand of the processed dsRNA is discarded, while the antisense strand functions as a guide for the specific identification of the target mRNA, which is then eliminated by endonucleolytic cleavage [39,40,41,42].

So far, three RNAi pathways have been described to be related to the Ago protein family members: siRNA, microRNA (miRNA), and PIWI-interacting RNA (piRNA), all with lengths of approximately 21–24 bp [43]. It has been reported that siRNAs are completely paired to a particular complementary region of a target mRNA, whereas miRNAs are not; thus, miRNAs have advantages over siRNAs, as diverse siRNAs can be produced from a unique miRNA [44,45]. While siRNAs are derived from exogenous dsRNA or DNA, long hairpins, or aberrant transcripts produced by repetitive genome sequences, miRNAs are generated from predecessor transcripts, named primary miRNAs (pri-miRNAs), which are normally transcribed by RNA polymerase II [46]. Pri-miRNAs are processed in the cell nucleus by a Dicer-like protein, termed Drosha, to generate a distinctive stem–loop conformation of approximately 70 bp in length, called pre-miRNA [47]. Later, the pre-miRNA is translocated to the cytoplasm, where the loop end is eliminated through the activity of Dicer, producing a mature miRNA, which is a dsRNA fragment of ~22 bp [48]. Subsequently, the mature miRNA binds to the RISC complex and produces its silencing effect by either eliminating complementary mRNA or binding imperfectly to complementary sequences within the 3′ UTR of the mRNA target, resulting in a translational repression [36].

The interference or silencing of the gene expression can occur either via the post-transcriptional siRNA route, or at the transcriptional level through the miRNA cascade, in which small non-coding RNA (ncRNA) molecules are converted into miRNA fragments [47,49,50,51]. These miRNAs can control the gene expression at the post-transcriptional level through the action of Dicer2 and Ago2, but also can be recognized by nuclear proteins, such as Dicer1, which result in the production of siRNAs that can interplay with Ago1 in the formation of the RNA-induced initiation transcriptional silencing complex (RITS). RITS can interact with chromatin to modify its structure around the target gene, resulting in heterochromatic silencing by, for example, inducing the DNA methylation or histone post-translational modifications [5,52,53,54,55,56]. Key components of the RNAi system are summarized in Table 1. Moreover, another Ago family protein, Ago3, is also related to ncRNAs linked with the transcriptional pathways that regulate transposable elements [57].

In general, RNAi is considered as a system of gene silencing that is presumed to have evolved as a cellular defense mechanism to destroy undesirable nucleic acids (such as transposons and viruses) in invertebrates, fungi, and plants [58]. Nevertheless, this strategy is also widely used by many eukaryotic cells to control the expression of endogenous genes [59].

### 2.1. Entry of RNAi Effector Molecules into the Organism

Like any external molecule, an RNAi effector faces different cellular and physiological obstacles that can affect its stability, delivery, and effective dose; thus, affecting its intended action. For example, both reticuloendothelial and renal systems trap molecules with diameters of more than 100 nm; therefore, the size of the RNAi fragments must be ideally in the range of 20–100 nm [60]. Moreover, once the RNAi fragments get into the intended tissue, they will find another obstacle, represented by the endothelial coating of tissues that presents pores ranging between 4.5 and 25 nm [60,61].

Even if the RNAi fragments manage to avoid the nuclease activity of the diverse body systems, for example, the digestive system and other barriers, such as the serum and the extracellular matrix, both size and negative charge can exclude their cellular absorption [60,62]. Moreover, the different endolysosomal sections are another boundary that can contribute to degradation of RNAi fragments via the endosomal envelope. Therefore, it is important that the correct arrangement and delivery of these fragments facilitate endocytosis and avoid hydrolysis so that the RNA can be incorporated into the RISC complex [60,63,64].

Additionally, some cellular proteins, such as the SID-1 protein, a highly conserved dsRNA transmembrane channel, can influence the absorption of RNAi fragments [65]. SID-1 plays a key role in the selective and specific passive translocation of dsRNA between cells and is essential for the wide systemic response of the RNAi mechanism [30,31]. Overexpression of SID-1 can significantly increase the systemic effect of exogenous dsRNA and, therefore, enhance the RNAi response. Homologues to SID-1 have been described both in fish [66] and shrimp [31,65] species; thus, the overexpression of SID-1, together with the delivery of exogenous dsRNA, could greatly enhance the RNAi effect in those organisms. Additionally, it has been reported that although the capacity of SID-1 to transport dsRNA is independent of the length of the fragment used [67], longer dsRNA molecules have shown a more efficient silencing of target genes [30,68]. One explanation for this observation is that longer dsRNA fragments can be processed into multiple siRNAs by the action of Dicer, thus generating a much more diverse and effective pool of siRNAs for integration into the RISC complex, in comparison with shorter RNA molecules [22,68,69].

SID-1 is not the only channel for dsRNA introduction into the cytosol. In other cases, both endocytosis-mediated pathways and class C scavenger receptors have been described as facilitators of dsRNA internalization, and these seem to be evolutionarily conserved [8,32,42,69,70]. For example, a class B scavenger protein (Croquemont), which has been reported in *Marsupenaeus japonicus* [71] and *Penaeus vannamei*, and the clathrin-mediated endocytosis mechanism, are also responsible for controlling the capacity to absorb dsRNA into cells [72,73].

### 2.2. RNAi Biogenesis in Shrimp

As in many eukaryotic species, the silencing pathways discussed above are well conserved in crustaceans and have key functions not only in transcriptional and post-transcriptional regulation, but also in responses against viral pathogens and the elimination of transposable elements [42,74,75].

In the case of penaeid shrimp, the Ago family members, key elements in the formation of RISC, were the first characterized proteins of a putative RNAi pathway [76]. It has been reported that shrimp Ago1 and Ago2 are linked to the dsRNA-mediated gene-silencing pathway [77], while Ago4 was revealed not to be associated with the RNAi machinery and the response against viral pathogens [45,78].

In *Penaeus monodon*, two isoforms (PemAGO and PmAgo) have been characterized [76,79], whereas *P. vannamei* possesses two different Ago genes (*PvAgo1* and *PvAgo2*) [65]. These genes are ubiquitously expressed, and their proteins were revealed to have the characteristics of the Ago protein family: a PAZ domain linked to PIWI and dsRNA-binding domains that have been previously shown to have RNase activity, thereby indicating their involvement in the RNAi machinery [65,76,79]. The silencing of the expression of *PemAGO* in *P. monodon* was shown to lower the capacity of dsRNA-mediated knock-down of an endogenous gene (nearly 50% of the original activity) [79]. Based on this finding, it has been proposed that other Ago subfamily proteins of penaeid shrimp could be linked to this system. In support of this hypothesis, the levels of expression of *PvAgo1* and *PvAgo2*, following dsRNA injection into shrimp, showed a robust response for *PvAgo2* only [65], whereas *PvAgo2* was associated with *PvDcr2* and *PvTRBP1* [80]. This indicates that the two Ago isoforms reported in *P. vannamei* can exercise specific roles, with PvAgo2 more directly related to the ‘siRNAi pathway’ [74].

Currently, a single Dicer gene has been described in *M. japonicus* and in *P. monodon* (*MjDcr1* and *PmDcr1*, respectively), whereas two Dicer-like genes (*PvDcr1* and *PvDcr2*) have been characterized in *P. vannamei* [80,81,82]. More recently, Shpaka et al. [42] reported both Dicer and Ago protein families in the giant freshwater prawn (*M. rosenbergii*). Phylogenetic and structural studies of the penaeid shrimp Dicer group have revealed that *MjDcr1*, *PmDcr1*, and *PvDcr1* encode putative proteins of 2473 and 2482 residues, with more than 95% similarity and grouping with the Dicer-1 type related to other vertebrates and invertebrates. On the other hand, the putative protein encoded by *PvDcr2* is 1502 residues and shows just 30% similarity with shrimp *Dcr1*, clustering with the Dicer-2 group reported in cnidarians and insects. In the case of invertebrates, such as shrimp, more than one Dicer has been characterized, and these have diverse roles and functional activities in the RNAi mechanism [82,83,84]. In *P. vannamei*, the characterization of two Dicer and Ago paralogues, combined with the fact that only *PvAgo2* expression was reactive to dsRNAs, has led to the notion that a ‘miRNA pathway’ is also functional in shrimp [65].

Additionally, the Dicer group has been shown to function in coordination with diverse dsRNA-binding proteins, such as a protein activator of PKR (PACT), and the HIV-1 transactivating response (TAR) RNA-binding protein (TRBP) in humans [85,86,87]. Other associated proteins, such as the eukaryotic initiation factor 6 (*eIF-6*), have also been characterized as a component of the RISC [88]. Diverse novel elements of the TRBP group have been reported in *M. japonicus* (*Mj-TRBP1-3*) and *Penaeus chinensis* (*Pc-TRBP1-3*) and present more than 99% identity [89,90]. A TRBP homologue was also characterized in *P. vannamei* [80]. Phylogenetic studies from several species show that these shrimp TRBPs are very conserved, in comparison to other elements of the same group, mostly in relation to dsRNA-binding domains. Homologues of *eIF-6* have also been characterized in *M. japonicus* and *P. chinensis* (*Mj-eIF6* and *Pc-eIF6*, respectively) and shown to be very similar in sequence to those reported in other species [89,90]. As in previous reports for other organisms [88], shrimp TRBP and eIF-6 showed a direct relationship, suggesting their interaction with the RISC [74].

**Figure 2:**
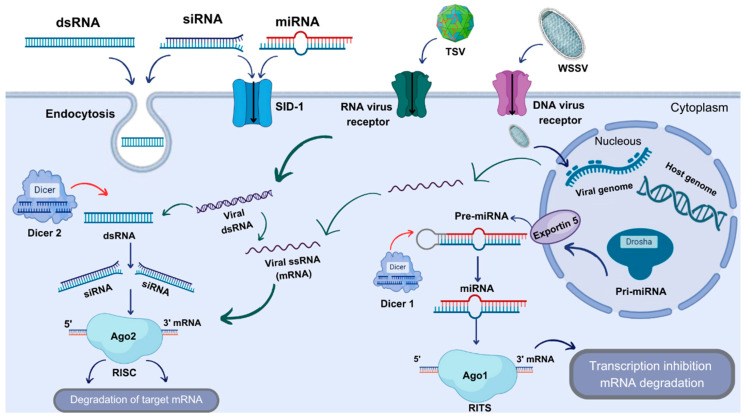
Schematic representation of RNAi pathways (exogenous and endogenous), including siRNA (RISC) and miRNA (RITS) pathways. Different RNA effector molecules (dsRNA, siRNA, and miRNA) are delivered into the cells (gills, hemocoel, intestine lumen, and hepatopancreas) through viral receptors, SID-1, and/or by clathrin-mediated endocytosis.

Furthermore, homologues of two proteins related to miRNA biosynthesis (PvPasha and PvArs2) have been reported in *P. vannamei*, showing, in both cases, more than 40% similarity with their insect orthologues. PvArs2 interacts with PvDcr2 and PvPasha [91]. Emerging information indicates, as in the case of other arthropods [92], that diverse small RNA regulatory pathways are functional in shrimp [74] (Figure 2).

### 2.3. Anti-Viral Silencing by RNAi

RNAi-mediated gene silencing was characterized in shrimp initially by Robalino et al. [93,94], who showed that in vivo delivery of dsRNAs in *P. vannamei* provided protection against viral pathogens and, therefore, revealed that these organisms exhibited an inducible anti-viral immunity against virus-related molecular structures. The notion of using RNAi as a viral management tool for shrimp was started in 2005 by diverse research groups, who showed that administration of long dsRNA notably enhanced the survival of shrimp challenged with a lethal dose of viral pathogens through the silencing of specific viral target genes [24,68,69,94]. Such a strategy has been successfully applied against all the main viral pathogens that affect the crustacean aquaculture industry; thus, the concept of dsRNA-mediated protection is well established [5].

In what constitutes a very powerful strategy, the RNAi mechanism can produce a specific and intense repression of virus replication in shrimp. Furthermore, the RNAi mechanism can be triggered either by siRNA or through miRNA. In the first case, the siRNA-related RNAi cascade is mediated through Dicer2 and Ago2, as viruses produce dsRNA as part of the replication process. This pathway is a very efficient mechanism to cope with infection from RNA and DNA viruses. In the second case, the miRNA-related RNAi pathway is controlled by Dicer1, Ago1, and Drosha, and produces a profound effect on multiple responses, such as host phagocytosis, autophagy, apoptosis, and over the virus latency/infection process, the modulation of host–virus interactions [95].

Key proteins linked with the RNAi pathway, such as Dicer and Ago, have been characterized in *P. monodon* [76,79,81], *M. japonicus* [74], and *P. vannamei* [80,82], thus supporting the presence of an RNAi system in shrimp. According to this, genes from important shrimp viruses, such as Taura syndrome virus (TSV) [96], yellow head virus (YHV), and white spot syndrome virus (WSSV), among others, have been silenced by the administration of sequence-specific dsRNA, thereby enhancing shrimp survival and suppressing viral replication [5]. Various reports have described the successful repression of shrimp viral infections by dsRNA, indicating that dsRNA can trigger an anti-viral immune response in crustaceans through two routes: one mediated by sequence-dependent dsRNA [26,97,98], and another through sequence-independent dsRNA [24,68,93,94,99].

One unexpected observation that has been described in many cases is that non-specific, non-target dsRNA, which has been employed as a negative control in viral silencing challenges, had a therapeutic effect. This has been reported not only in the case of YHV [24], but also in challenges against WSSV [94], by the induction of non-specific immunity [69,93,100]. Furthermore, in the case of *P. monodon*, even totally unrelated, non-specific dsRNA, such as that from the green fluorescent protein (GFP) gene sequence, can silence the YHV genes [24]. Likewise, La Fauce and Owens [101] described a non-specific dsRNA-silencing effect against *Penaeus merguiensis* Densovirus (*Pmerg*DNV).

In the case of shrimp, Robalino et al. [94] suggested that the systems that trigger the dsRNA-induced innate immunity and the RNAi mechanism could interact functionally during the anti-viral response, enabling the host defense against these pathogens [94,102]. In support of this assumption, it was later proved that in the case of *P. vannamei*, unrelated dsRNAs induced the expression of *PvAgo2*, indicating the presence of a potential link or functional overlap among the non-specific stimulation of the anti-viral immunity and the RNAi system [65,74].

Nevertheless, despite the observation of the benefits from the administration of unspecific exogenous dsRNAs in shrimp, the sequence-specific dsRNAs have shown to be the best option for viral disease management, as this leads to a more robust anti-viral reaction, in comparison to non-specific dsRNA, especially in challenges with higher viral loads [94]. Furthermore, in *P. vannamei*, it has been reported that the non-specific effects of unrelated dsRNAs are dependent on the molecule length, as only dsRNAs of more than 50 bp, and not siRNAs, can generate a defense against viral pathogens [65,74,94]. Moreover, in comparison with shorter strands, longer dsRNA induces the production of a highly diverse pool of siRNAs that can be integrated into RISC [68]. Thus, a precise design of RNAi effectors, as well as the implementation of proper controls, should be used when testing RNAi-related knock-down in crustaceans [5].

### 2.4. The siRNA-Mediated Anti-Viral Immune Response

From an evolutionary perspective, RNAi triggered by siRNA is a very conserved anti-viral mechanism in most eukaryote organisms [103]. The expression silencing through siRNA has been widely reported many times in shrimp [65,90,104,105], and both siRNA and long dsRNA can inhibit viral multiplication, including the cases of infectious myonecrosis virus (IMNV), *Penaeus monodon* Densovirus (*Pm*DNV), YHV, WSSV, and TSV [8,24,26,97,98,106]. For example, in *M. japonicus*, siRNA-mediated RNAi has been described as a relevant immune defense approach against virus pathogens involving the elimination of viral mRNAs [97]. The siRNA-induced anti-viral system is supported by the fact that some viruses produce dsRNA during their replication process [107]. During infection with RNA viruses, the viral genomic RNA is reproduced in the host cells and then is modified to produce dsRNA precursors. Later, this dsRNA is converted via Dicer2 to virus-derived siRNAs, which are integrated into the RISC by Ago2, leading to degradation of target viral RNAs and, therefore, inhibition of viral replication [108].

It has been reported that siRNA presents a seed area (2nd–7th nt) and a supplementary zone (12th–17th nt) [109]. The seed zone is related to the first phase of identification of the target, whereas the supplementary zone facilitates the target-binding process. These data highlight the relevance of the siRNA-induced RNAi immune response of shrimp against RNA virus infections. Additionally, throughout DNA virus infection, viral mRNA fragments could generate molecules with double-stranded structures, for instance, an mRNA hairpin, that can be identified and processed through Dicer2 [110]. The produced duplex siRNA is integrated into RISC, leading to viral mRNA elimination, which can inhibit the virus replication [111]. For example, it has been reported that *M. japonicus* can produce an anti-viral siRNA, named siRNA-VP28, to cope with WSSV infections. Throughout this process, Dicer2 and Ago2 are needed for the generation and functionalization of the siRNA-VP28 [95,110].

Both long dsRNA and siRNA can be used to cope with viral pathogens. As mentioned above, a dsRNA molecule can be transformed into diverse siRNAs in vivo; however, it is not clear which siRNA molecules exert the effect. Thus, those multiple siRNAs could impact the expression of other genes besides the target gene [9].

### 2.5. The miRNA-Mediated Anti-Viral Immunity

Increasing data show the fundamental function of miRNAs in the process of gene regulation in eukaryotes, and some authors have characterized how miRNAs can affect the innate immune response in crustaceans. For example, during a WSSV challenge, miR-7 can target the 3′ UTR of the early gene *wsv477*, which implies that miR-7 is related to viral DNA multiplication [112,113,114]. Therefore, the immune response of crustaceans is heavily controlled through miRNAs. The miRNAs are key factors related to the regulation of a wide range of biological functions, such as immunity, apoptosis, metabolism, cell differentiation, proliferation, and development [115]. The silencing of target genes mediated by miRNA has the features of a control network, which implies that a particular miRNA can not only target several genes at the same time, but also that different miRNAs can target the same gene [116,117,118,119,120].

In *M. japonicus*, throughout the virus–host interaction, a particular and specific host miRNA can induce simultaneous mRNA elimination for several different viral genes, and different viral-derived miRNAs can simultaneously target the same mRNA [116,120]. For instance, *M. japonicus* miR-12 can simultaneously induce anti-viral immunity, apoptosis, and phagocytosis by targeting shrimp genes, such as the phosphatase and tensin homolog (*PTEN*), transmembrane BAX inhibitor motif containing 6 (*BI-1*), and viral genes, such as *wsv024* [121]. In fact, miRNA may be the link between viral infection of shrimp and cellular autophagy [122]. These control mechanisms are shown to be very important during virus latency/infection and for host phagocytosis, autophagy, and apoptosis, which eventually have a deep influence over the host–virus interactions. Furthermore, shrimp apoptosis can be controlled via miR-100, which has been shown to have an anti-viral role [9,123].

Beyond the seed region (2nd–7th nt), the non-seed sequence (9th–18th nt), which is complementary to the target mRNA sequence, is fundamental for miRNA targeting [122,124]. In the case of animals, such as shrimp, miRNA can induce the digestion (exonucleolytic 5′–3′ activity) of its target mRNAs, which stops at sites within 3′ UTRs of target mRNAs, near the zone complementary to the miRNA seed region [95].

Moreover, it has been reported that WSSV can encode forty different viral miRNAs, such as miR-212 and miR-211, which contribute to the WSSV infection [118]. Around 63 shrimp miRNAs were recognized, of which 6 were downregulated, while 25 were overexpressed in response to WSSV [113]. Furthermore, 24 miRNAs linked to the pro-phenoloxidase system, apoptosis, and phagocytosis were also identified [125]. Between the 24 innate-immune-related miRNAs, 21 were very conserved, indicating that these miRNAs share the same or similar roles in diverse animal species [9,125].

Generally, the locations of miRNA genes are well conserved, typically located within introns [126]. Currently, thousands of miRNAs derived from plants or animals are reported on miRbase, including different species of aquatic crustacean [127]. There is a significant body of information that points to the fundamental functions of the miRNAs in the process of anti-viral regulation of aquatic organisms [9]. Moreover, it has also been reported that shrimp miRNAs can improve apoptosis and phagocytosis by targeting particular genes, triggering the inhibition of viral progression [9,121,128].

## 3. Use of RNAi for Controlling Other Non-Viral Pathogens

### 3.1. RNAi for the Control of Bacterial Pathogens in Shrimp Aquaculture

In the shrimp aquaculture sector, the second most important group of pathogens after viruses, in terms of their incidence and the economic losses caused, are bacterial species, mainly represented by those from the *Vibrio* genus [129,130]. Vibriosis, caused by species such as *V. vulnificus*, *V. anguillarum*, *V. harveyi*, *V. alginolyticus*, and *V. parahaemolyticus*, has been regularly identified among cultured shrimp [129,130].

The use of RNAi technology has also been tested to inhibit the replication of this type of pathogen in shrimp aquaculture. For instance, Peng et al. [131] reported that silencing of the *PvGRIM-19* gene via dsRNA led to a lower level of mortality in *P. vannamei* infected with *V. alginolyticus*, as well as a higher level of expression of STAT and JAK genes. More recently, Zuo et al. [132] reported that silencing of a JAK-STAT cascade, targeting the endogenous *Ftz-F1H* gene (*Fushi tarazu* transcription factor), improved the resistance of *P. vannamei* against *V. parahaemolyticus*, as both the rate of mortality and bacterial load were significantly lower after dsRNA-mediated knock-down. *V. parahaemolyticus* is the etiological agent responsible for acute hepatopancreas necrosis disease (AHPND), black gill disease, and red leg disease [133,134,135]. Furthermore, in the *Ftz-F1H*-knock-down shrimp, the levels of expression of a group of immune effectors and the hemocytes and phagocytic activity were significantly higher [132].

Moreover, Huang et al. [136] reported that dsRNA-mediated knock-down of the *DJ-1* gene in *P. vannamei* (*PvDJ-1*) led to an increased level of protection against *V. alginolyticus* infection, as cumulative mortality in the *PvDJ-1*-silenced shrimp was significantly lower compared to the control after a *V. alginolyticus* challenge. Such data suggest that *PvDJ-1* regulates antioxidant and apoptosis activities, thus playing a key function in *P. vannamei* defense against *V. alginolyticus* [136]. Similarly, Li et al. [137] reported that targeting the cytochrome c4 (*cyt-c4*) gene of *Aeromonas hydrophila* through short-hairpin RNA (shRNA) led not only to a decrease in biofilm formation but also a lower level of pathogenicity and drug resistance of *A. hydrophila*, suggesting that *cyt-c4* serves an important function in both biofilm generation and virulence of *A. hydrophila*.

### 3.2. RNAi for the Control of Shrimp Parasites

The parasite microsporidian, *Enterocytozoon hepatopenaei* (EHP), is another important pathogen that heavily affects the shrimp aquaculture sector worldwide. Acute EHP infections produces both slow growth and higher susceptibility to other opportunistic pathogens [138]. EHP generates spores of chitin walls in order to survive during prolonged environmental exposure. Moreover, polar tube extrusion is essential for EHP infection [139]; therefore, the inhibition of extrusion can prevent EHP infection. Indeed, dsRNA-mediated silencing, targeting the polar tube protein 2 of EHP (EhPTP2) via injection, significantly reduced the EHP copy number in infected *P. vannamei*. Furthermore, the delivery of this specific dsRNA into EHP-infected *P. vannamei* prior to their use in cohabitation with naïve shrimp significantly reduced the rate of EHP transmission [140].

## 4. Design of RNAi

### 4.1. Factors Influencing RNAi Molecule Design and Silencing Effect

Generally, the design of dsRNA effector molecules starts with the implementation of standard algorithms to characterize the RNA secondary structure and to avoid off-target effects that might result in the unwanted silencing of host genes. Identification of potential off-targets can be accomplished by subjecting potential target sites to BLAST searches [141] using available host transcriptome and genome sequences [8].

Moreover, diverse parameters, including the target pathogen [142,143], administration frequency [144], transfection reagents [145], delivery route [146], dosage [147,148,149,150], target tissue [151,152], length of the fragment [26,65], RNAi effector molecule [94,145], nucleotide sequence [97,153], and selected target gene [68,94,99,144,154], can influence the success of the implementation of the RNAi technology applied to aquatic farmed animals.

The anti-viral response triggered by RNAi can be dependent not only on the target [25,68,94,99,144,155], but also on the dose [24,156,157]. For instance, the administration of RNAi targeting non-structural viral genes, such as those encoding the helicase, protease, DNA polymerase, ribonucleotide reductase small subunit (*rr2*), thymidine, or thymidylate kinase, has been consistently reported as the more efficient target, in comparison to targeting structural genes. This fact has led to the suggestion that the silencing of low-expressed genes would be a better approach to inhibit viral replication [5]. Nonetheless, despite this strategy being adopted in several cases [25,68,94,155,158], the hypothesis must be taken with caution, as different reports have indicated that RNAi targeting viral structural proteins might also confer strong protection against viral replication [99,159,160,161,162].

Thus, the data do not show any specific predictability in the target choice based on the virus gene type (non-structural or structural) and do not necessarily support the suggestion that non-structural genes are better targets for RNAi, because they typically have low expression levels, in comparison to structural genes [155]. For example, different RNAi effector molecules targeting the VP28 or VP19 structural proteins of WSSV have been shown to strongly suppress viral replication, even though these genes are higher expressed compared to the non-structural genes [163]. Nevertheless, in other cases, such as IMNV, the knock-down by RNAi of the non-structural RNA-dependent RNA polymerase (RdRp) was much more effective than the targeting of capsid proteins [164].

According to the data currently available, it is possible to conclude that there are not any evident criteria to predict the RNAi efficiency based on the viral gene targeted. The RNAi approach has been successfully used to inhibit the viral replication not only by silencing genes that are high or low expressed, but also at the start or at the end of the infectious process. Thus, the prediction of a more efficient target gene cannot be made based on the protein function [8]. Beyond the virus gene category, other researchers have suggested that some genes are differentially influenced by RNAi. Moreover, the efficiency of the RNAi design may be influenced by whether the target gene is needed for viral pathogenicity [94].

On the other hand, the dose of dsRNA needed to stimulate the RNAi response and then inhibit viral replication does not seem to be very important, maybe due to the RNAi guide being reused and reintegrated into the RISC. In the case of crustaceans, the doses have ranged from two doses of 0.5 to 7.5 μg/g, with a mean of 2.6 μg/g (SD = 2.27 μg/g, n = 10) [165].

The RNAi efficiency can also be influenced by the length of the RNAi molecule used. Different groups have reported that small molecules, such as siRNAs (21 bp), designed against viruses showed a weak protection effect. Robalino et al. [94] pointed out that siRNAs are not good inducers of the sequence-specific anti-viral immune response and stated that they are unable to generate an anti-viral effect. For instance, a full-length VP19 dsRNA showed a strong level of viral inhibition, in comparison to a siRNA targeting the same gene. Moreover, a siRNA targeting *rr2* led to only 50% protection [97], whereas a long dsRNA provided 88% of the protective effect against WSSV [94].

In the case of IMNV, RNAi effector molecules can be as short as 81 bp, providing 100% protection; nonetheless, shorter molecules (54 bp) showed a sub-optimal efficiency. All these data have led to the hypothesis that there is a minimum-length threshold needed for the successful induction of the anti-viral response [164]. This suggestion is supported by other reports that revealed that only dsRNA greater than 50 bp, and not siRNA, were able to knock-down native genes or stimulate the expression of the RNAi pathway genes [65].

More recently, Shpak et al. [42] showed that in vivo silencing by RNAi is length dependent. The use of long dsRNA was shown to be more efficient, while siRNA did not reveal any effect. Despite previous reports that illustrate that small dsRNA molecules can trigger a RNAi response in vitro, the length dependency demonstrated in vivo suggests a more complex mechanism in the context of the live organism [42]. Therefore, long dsRNA is more efficient in targeting specific genes in the case of crustaceans [68]. Likewise, similar results have been reported in the case of other invertebrates, such as *D. melanogaster* and *C. elegans* [34,35]. On the other hand, the administration of dsRNA in a non-specific manner only produces a short-term and low-level protection in the case of WSSV, but not for IMNV [164]. Thus, the delivery of unspecific RNAi led to an improved shrimp anti-viral immune response but failed to produce virus-specific protection and did not provide a general anti-viral effect [8].

### 4.2. Simultaneous Silencing of Host and Pathogen Genes

While most of the initial studies about the use of RNAi in shrimp were focused on the protection against viruses by knock-down of viral genes through intramuscular injection of sequence-specific dsRNA [5], dsRNA targeting host genes has also been applied, and this approach was shown to confer the additional benefit of a multivalent effect of protection. For instance, the knock-down of the shrimp endogenous gene *Rab7* produced a simultaneous protection effect against YHV, WSSV, TSV, and Laem Singh virus (LSNV) [166,167,168,169]. This multivalent effect was likely due to the fundamental role of Rab7 in the host endocytosis cascade that is used by all these viruses throughout the infection cycle. However, Rab7 is also related to other key cellular mechanisms; thus, there are some concerns about the potential negative side effects that may arise on the shrimp’s health if this gene is silenced for a long period of time [69].

The silencing of other host genes linked to pathogen uptake and replication, such as virus-binding proteins and cell surface receptors, has also been extensively studied. Among these, the most frequently explored were the lymphoid cell-expressed receptor [170], Laminin receptor protein (Lamr) [171], β-integrin [172], hypoxia-inducible factor-1α (HIF-1α) [173], and Rab/Ras GTPase proteins [166,167,168,169,174,175,176,177]. Moreover, if it is considered that the mechanism of viral uptake into the cells is controlled through both viral and cellular factors, the simultaneous delivery of RNAi targeting host and viral genes may potentially produce an enhanced protection effect compared to the administration of the same constructs separately. This hypothesis was corroborated by Posiri et al. [178], who reported that a single injection of a mix of dsRNAs, targeting both the YHV protease gene and the endogenous *PmRab7*, led to a significantly reduced mortality compared to dsRNAs injected individually [24,26].

Likewise, the simultaneous knock-down of the WSSV immediate-early protein (IE1) and the *P. monodon* TATA box-binding protein (PmTBP) led to a substantial reduction in the viral load, in comparison to when each gene was individually knocked-down [179]. Interestingly, the same effect was not reported by Attasart et al. [144] when they tried to simultaneously silence the expression of WSSV *rr2* and *PmRab7*. Several injections of either mixed dsRNAs or dsRNA-*rr2* alone similarly enhanced the survival rates by around 95%, whereas only a partial effect was conferred by a non-specific dsRNA (GFP) and dsRNA-*PmRab7* (approximately 55%) [144]. The differences between these studies can be attributed to the differences in shrimp sizes, target genes, dsRNA ratios, injection frequencies, and viral loads implemented in each case. Thus, despite the theoretical potential of simultaneous silencing as an enhanced approach to the management of shrimp viral infection, it seems that a detailed optimization of several variables is needed to achieve consistent results [5].

### 4.3. Capacity of RNAi to Generate a Prophylactic and/or Therapeutic Effect: Dose and Timing of Prophylactic Administration of RNAi

Many studies have revealed that delivery of dsRNA/siRNA targeting a specific pathogen before or simultaneously to a viral challenge can lead to inhibition of the replication of diverse virus types [68,97,146,180]. Furthermore, in the case of shrimp, it has also been demonstrated that the administration of RNAi effector molecules to pre-infected shrimp can also produce a therapeutic response [26,98,155,178,181]. Such curative effect can be highly enhanced by the simultaneous silencing of not only structural and non-structural viral proteins [98] but also of host genes [178]. However, therapeutic efficiency depends on several factors, such as the duration of pre-infection, which usually had lower efficiency when the length of dsRNA administration after infection was longer. This therapeutic effect can be very useful in hatcheries, particularly to avoid the loss of valuable broodstock by viral infections. Such a strategy has shown its high impact in the clearance of non-lethal LSNV in *P. monodon*, which has infected almost 100% of broodstock in Thailand and India [182,183].

Nevertheless, due to the short lifetime of the dsRNA-silencing effect, the possible implementation of a prophylactic mode of administration at the grow-out cycle would need repeat dosing of the shrimp over long periods, and this leads to a higher cost. Therefore, the therapeutic use of this technology during a short period to delay the progression of an outbreak might be a more viable alternative (allowing the time necessary for an emergency harvest, for example). Moreover, it could also be implemented for a short period in a hatchery in the case of shrimp infected at a vulnerable life stage to help them reach a more resistant life stage. Thus, the potential for field applications will be related not only to the cost, but also to the objectives and points of application in the shrimp-farming cycle [69].

Additionally, the timing of the administration of the dose in the prophylactic mode is more important, as several authors reported a loss of activity against viruses with the passing of time. Indeed, most reports pointed out that the administration of RNAi at 24 h before the virus challenge yielded the best result. In some cases, a protective effect against YHV, for example, was seen up to four days after the virus challenge [24]. Nonetheless, some reports highlighted that administration of dsRNA 24 h after a YHV challenge was admissible, but not 48 h after challenge [178]. Normally, prophylaxis is better than therapeutic treatment; however, in this case, the treatment is feasible right after potential virus exposure.

### 4.4. Synthesis of RNAi Effector Molecules

At the research level, long dsRNA molecules can be synthesized either through in vivo or in vitro transcription procedures. In the case of the second method, the transcription can be carried out by specific RNA polymerases to synthesize sense and antisense RNA strands using a linearized vector that has an RNA polymerase promoter, such as that from SP6, T7, or T3 bacteriophage. Subsequently, and through a gradient of temperature, both complementary strands are annealed to produce dsRNA [184]. Alternatively, ssRNA can be used as a template for a RdRp, such as the Φ6 RNA polymerase, to synthesize dsRNA. Subsequently, remanent ssRNA can be eliminated through precipitation with lithium chloride to obtain dsRNA [185]. Alternatively, templates generated by PCR containing T7 promoters at both ends may also be employed to produce dsRNA by in vitro transcription [69]. Likewise, there are currently available protocols for cost-effective in vitro production of siRNA [186].

On the other hand, production of dsRNA in vivo involves engineered cells that transcribe a particular DNA sequence that previously was cloned into a vector or into the host chromosomal DNA. In this case, the most employed methodology is based on the expression of dsRNA using bacterial platforms. Most of the reports about the use of dsRNA applied to shrimp used *Escherichia coli* strain HT115 (DE3) [187], which is deficient in ribonuclease III activity, thus allowing the accumulation of higher levels of dsRNA within the cells, as it is not degraded by the action of this enzyme. For large-scale production, such a strain can be used to produce dsRNA using the T7 promoter [188,189]. Furthermore, other methods are available, including the use of *Pseudomonas syringae* harboring a Φ6 polymerase complex [185].

Although bacteria-mediated methods are still generally employed to generate dsRNA [190], microalgal expression platforms are gaining more interest, mainly due to the rising concerns related not only to the potential risks linked to the spread of antibiotic resistance genes in the environment [191], but also the rejection of such bacterial expression systems by consumers [69]. In this regard, the pioneering report of Somchai et al. [192], in which they described the successful transformation of the harmless green microalga *Chlamydomonas reinhardtii*, employing a vector that produced dsRNA against YHV, opens new possibilities for the application of this expression platform in shrimp aquaculture. Moreover, the selection of the transgenic strain of *C. reinhardtii* (expressing specific dsRNA) could be performed without the use of an antibiotic resistance gene as a selection marker [193].

Recently, Alvarez-Sanchez et al. [194] reported the generation of dsRNA (targeting the WSSV *ORF89* gene) employing yeast cells (*Yarrowia lipolytica*). In this case, the yield of dsRNA synthesized through the engineered strain (P01-AS) reached 182 ng/L of culture. Nonetheless, different reports have revealed that only a few nanograms of a particular dsRNA can lead to the degradation of the target mRNA, thus triggering a significant reduction in the viral copy number [193]. These findings highlight the potential of this approach as an oral therapeutic strategy against WSSV in shrimp aquaculture.

More recently, Rattanarojpong et al. [195] reported that a genetically modified baculovirus mediated dsRNA administration and led to a protective effect against WSSV. According to these authors, shrimp injected with a recombinant baculovirus harboring dsRNA targeting *VP28* and *rr2* revealed a significantly lower cumulative mortality of 33% at 14 dpi, in comparison to the animals only treated with baculovirus harboring *VP28* dsRNA alone, in which the level of cumulative mortality reached 64%. Such results pave the way for the use of bacterially (*E. coli* DH10Bac^TM^, Life Technologies Corporation, CA, USA) expressed recombinant baculovirus mixed with shrimp feed for the control and prevention of WSSV.

## 5. Delivery of RNAi Effector Molecules

**Figure 3:**
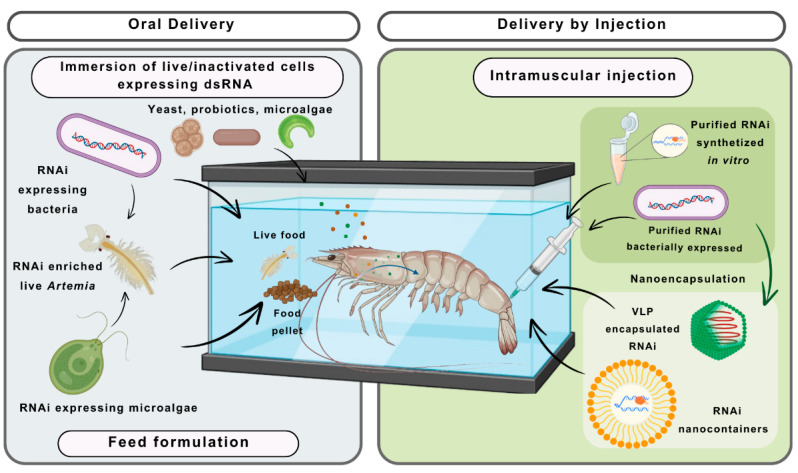
Potential routes of production and delivery of dsRNA to farmed shrimp.

RNAi has been used to further basic research by characterizing the role and molecular mechanism of a wide group of crustacean genes. Moreover, RNAi also represents a strategic tool to investigate the cellular mechanism linked to the anti-viral defense system of commercially important, non-model crustaceans [5]. Generally, the administration of dsRNA through injection is the most used strategy to study the silencing of host and viral genes at the laboratory level, where the cost is not a limiting factor. Additionally, there are other cost-effective options, such as oral delivery, that can be applied in shrimp broodstock facilities to supply post-larvae (Figure 3). However, no practical approach has been developed for the administration of dsRNA at the farm level. So, to boost the use of RNAi applied to shrimp, a more efficient method of administration must be developed [45].

These methods will depend on several factors, such as the study goal, target cell type, and the mechanism to reach the target. Additionally, the efficiency of the RNAi approach relies on the stability of RNAi effector molecules throughout the administration process: during and after. Therefore, at the farm scale, two fundamental challenges need to be overcome to successfully apply RNAi: (i) the development of a cost-effective pipeline for the generation of dsRNA, and (ii) an oral administration procedure that can deliver and protect the effector dsRNA molecules to the infected host cells [45,69].

### 5.1. Oral Administration of RNAi Effector Molecules in Shrimp

Oral delivery by the inclusion of an RNAi effector in the shrimp feed is the most effective and preferable approach used. Among these, the use of dsRNA expressed by bacteria or microalgae, or a live feed of *Artemia* (pre-fed with bacteria expressing the dsRNA), are the methods that allow efficient administration of dsRNA. Alternatively, both biological and non-biological nano-containers have been explored, with promising results. Those nano-containers provide an attractive option to improve the lifetime of the dsRNA and the delivery into the target cells [69]. Such nano-containers include virus-like particles (VLPs), liposomes, and chitosan nanoparticles, among others, as additives for food pellets applied to pond-reared and/or hatchery shrimp [45,69].

#### 5.1.1. Oral Delivery of Bacterially Expressed dsRNA

Different approaches have been explored for oral delivery of bacterial cells expressing dsRNA. Sarathi et al. [159,160] were the first group that reported the possibility of inhibiting WSSV replication in *P. monodon* by the ingestion of food pellets coated with formaldehyde-inactivated *E. coli* expressing VP28-dsRNA. Nevertheless, in comparison with injection, lower protection was accomplished with this oral delivery system, which can be explained by the degradation of dsRNA molecules throughout the oral delivery process. Moreover, in the case of inactivated bacteria expressing dsRNA against WSSV, the reduction in the rate of mortality was almost double that achieved with nanocarriers (chitosan dsRNA complex) [159], which suggests that oral administration using bacteria is considerably more efficient than nano-containers. One possible explanation is that inactivated bacteria may provide higher protection for the dsRNA and could be more efficient in crossing the shrimp gut, in comparison with the chitosan nanoparticles. In contrast, 100% mortality was reported in the control group (shrimps fed with inactivated *E. coli* transformed with the empty plasmid). Furthermore, simultaneous cultivation of two different RNase-deficient *E. coli* strains using Terrific Broth medium can significantly increase the yields of dsRNA obtained. Importantly, the optimal production conditions of the dsRNA were achieved by glycerol fermentation, making this approach suitable for industrial-scale applications [45,182]. The strategy of coating feed pellets with bacteria expressing dsRNA was also described in the case of *P. monodon* for the control of slow-growth syndrome caused by LSNV [183]. Interestingly, these researchers concluded that, at least in the case of slow-growth syndrome in *P. monodon*, the enhanced weight gain could justify the additional cost due to the administration of dsRNA.

Furthermore, Leigh et al. [196] studied the comparative therapeutic effect of dsRNA against *Penaeus merguiensis* Hepandensovirus (*Pme*HDV) using *E. coli* transformed with a specific and a non-specific RNAi vector. A higher survival rate was reached with live *E. coli* (68%), whereas for the other conditions (specific RNAi, non-specific RNAi, and naked vector), a similar lower level of protection was accomplished. Additionally, the use of a formulated diet has been reported based on inactivated bacteria transformed with vectors for the expression of dsRNA-vp (structural capsid protein) and dsRNA-ns1 (non-structural protein 1), targeting *Pm*DNV [197]. These authors showed that in the prophylactic mode, the level of viral suppression was 88%, while in the therapeutic mode it was 64%. On the other hand, in the case of *P. monodon*, Sellars et al. [146] described that oral delivery of dsRNA generated by bacteria, and targeting the gill-associated virus (GAV), enhanced survival following intramuscular injection, but not when the dsRNA was orally administered. These authors suggested that some of the factors that contributed to these results are related to an insufficient dsRNA absorption through the gut wall, and to dsRNA elimination by the nucleases present in the digestive tract of shrimp.

Interestingly, it has also been reported that probiotic bacteria, such as *Lactobacillus plantarum* and *Bacillus subtilis*, can be exploited as alternative platforms to express specific dsRNA and perform dual functions of health improvement and anti-viral protection. *L. plantarum* producing dsRNA targeting the RdRp gene of YHV produced a suppression of virus replication when orally administered, without losing its inhibition effect against *V. parahaemolyticus* [198]. Similarly, *rnc* gene deletion in *B. subtilis*, which eliminated a type III ribonuclease, making the strain unable to degrade dsRNA, exhibited its capability in efficient expression of dsRNA targeting VP28 and protection from WSSV infection. The effect of dsRNA in the host was also detected through upregulation of *SID-1*, *Dicer2*, and *Ago2* [199].

Another approach for oral administration is the use of *E. coli* expressing dsRNA immobilized in agar (2%). In this study, dsRNA targeting the endogenous gene *Rab7* (dsRNA-Rab7) or the YHV protease gene (dsRNA-Pro) was used to inhibit YHV replication, achieving a significant reduction in shrimp mortality. Interestingly, the combination of dsRNA-Pro and dsRNA-Rab7 did not show an improved inhibitory effect, in comparison to the administration of dsRNA-Rab7 alone [200].

Generally, the use of RNase III-deficient *E. coli* strains (such as HT115) has been used for large-scale generation of dsRNA, as they are very efficient and inexpensive [168,190]; however, this approach can be problematic, as its long-term effects on the treated organisms and within the environment are poorly understood. Therefore, finding safer alternatives for the generation and administration of dsRNA is a key challenge that must be overcome for the future deployment of RNAi technologies.

In another interesting example, Naveen Kumar et al. [201] documented a protective effect in *M. rosenbergii* against white tail disease by the oral delivery of *E. coli*-expressed and encapsulated dsRNA (targeting *B2* and capsid genes of *Macrobrachium rosenbergii* Nodavirus (*Mr*NV) and a capsid gene of the extra-small virus—XSV). XSV is a satellite virus of *Mr*NV (classified as Macrobrachium satellite virus 1), as it depends on the RdRp of *Mr*NV for its replication [202]. Shrimp fed (using feed coated with inactivated *E. coli* cells) with a mix of dsRNA targeting *Mr*NV and XSV capsid genes exhibited a high survival rate (80% and 75% at 24 and 72 h, respectively), while 100% mortality was reported for the control group [201]. Furthermore, live or inactivated bacteria expressing a specific dsRNA can be used to feed aquatic organisms (e.g., *Artemia*); therefore, this strategy can be applied to provide a wide range of protection against viral pathogens [160,175,182].

#### 5.1.2. Bio-Encapsulation of dsRNA Expressing Bacteria into *Artemia*

The use of shrimp brine (*Artemia*) larvae has been extensively applied as live feed in the aquaculture industry, mainly because of its availability as commercial *Artemia* cysts. Due to its use with non-specific filter feeders, *Artemia* has become a very attractive platform for the enrichment or bio-encapsulation of a diverse range of high-value nutritional and/or therapeutic compounds. This enriched *Artemia* has been used to feed shrimp, fish, or other aquatic organisms in applications ranging from disease therapy, sex conversion, and induction of ovulation [203,204]. According to this approach, *Artemia* larvae are bathed in a suspension containing bioactive compounds, such as probiotics, recombinant proteins, or dsRNA-expressing bacteria, before their use as a feed [205,206]. Therefore, *Artemia-*enrichment technology can be a promising delivery method for the administration of dsRNA applied to shrimp aquaculture. For example, this approach has been used in the administration of *E. coli* expressing dsRNA targeting LSNV into post-larvae of *P. monodon* [183,207]. Furthermore, the use of *Artemia* enriched with *E. coli* producing red fluorescent protein (RFP) revealed a fluorescent signal in the intestine, which means that the bio-encapsulation within the bacteria was successful. Compared with the control group (post-larvae fed with *Artemia* enriched with normal *E. coli*), dsRNA-LSNV-enriched *Artemia* led not only to a significantly lower LSNV viral load but also to a body weight improvement. Considering several factors, such as feed attraction, post-larvae densities, and the small tank sizes, the *Artemia* enrichment approach represents an inexpensive and practical system for the control of viral pathogens that can be applied to hatchery shrimp aquaculture [69].

#### 5.1.3. Oral Delivery of Microalgae-Expressed dsRNA

The use of microalgae as a food ingredient is attractive, as it does not represent any health risk or environmental contamination. For these reasons, microalgae have been used as a functional feed ingredient for the administration of bioactive compounds, such as antibacterial additives, vaccine subunits, and virus-targeted RNAi molecules [193,208,209].

Genetic engineering procedures to express recombinant proteins or dsRNA using *Chlamydomonas* are well established and involve random DNA integration in the nuclear genome or targeted integration by homologous recombination in the chloroplast genome [192,210,211]. Moreover, the ‘Generally Recognized As Safe’ (GRAS) status of *C. reinhardtii* and the lack of production of any infectious agents or endotoxins makes it especially useful [193]. Strains of genetically modified *Chlamydomonas* have been used to generate immune-protective proteins to enhance the survival of shrimp and fish challenged with bacterial or viral pathogens [212]. Recently, Lanh et al. [213] used *C. reinhardtii* for nuclear expression of the WSSV capsid protein VP28. The oral delivery of this strain of *C. reinhardtii* improved the shrimp survival (70%), in comparison to the control group (0%). These results confirmed that genetically engineered *C. reinhardtii* expressing VP28 can be used as an oral treatment against WSSV [214].

Moreover, a similar approach using *C. reinhardtii* has been successfully applied in shrimp to produce dsRNA against YHV. In a seminal work, Somchai et al. [192] used *C. reinhardtii* for the expression of the YHV-specific hairpin RNA targeting *RdRp*. The oral administration of this engineered strain of *C. reinhardtii* to shrimp post-larvae improved the survival (22%), in comparison to a control (5%), following challenge with YHV.

Furthermore, Charoonnart et al. [193] modified the chloroplast genome of *C. reinhardtii* to express dsRNA targeting the YHV *RdRp*. Interestingly, these authors described the generation of a stable strain of *C. reinhardtii* without using an antibiotic resistance gene as a selection marker. The oral administration of this genetically modified *C. reinhardtii* strain improved the survival of shrimp (50% at 8 dpi) compared to the control group (84.1% mortality) after a challenge with the YHV. Moreover, their results showed a lower infection rate in the treated shrimp (55.6 ± 11.1%), in comparison to controls (88.9 ± 11.1% and 100.0 ± 0.0%, respectively). Importantly, the whole-cell dsRNA administration approach might not only enhance the dsRNA protection against environmental degradation but also offers additional nutritional benefits through *C. reinhardtii* consumption. Taken together, these results highlighted microalgae as a sustainable alternative for the synthesis and oral administration of anti-viral compounds applied to shrimp aquaculture [193].

Charoonnart et al. [215] reported the generation of anti-viral dsRNA in the *C. reinhardtii* chloroplast through a convergent promoter approach. In this case, approximately 100 µg of dsRNA was synthetized per L of genetically modified *C. reinhardtii*, which equated to a 10-fold increase compared to previous studies using convergent promoters [193]. In their more recent investigation [215], the promoter used belonged to the *C. reinhardtii* chloroplast gene *rrnS*, which encodes the 16S ribosomal RNA. This is the most highly expressed gene in the chloroplast of *C. reinhardtii* [216,217]. Furthermore, *P. vannamei* administered feed mixed with the freeze-dry transgenic strain of *C. reinhardtii* expressing dsRNA (targeting the VP28 gene of WSSV) showed a significantly improved survival (69%) against WSSV infection compared to the negative control, without RNAi treatment. The approach proposed by Charoonnart et al. [215] represents a promising enhanced microalgal production platform for the generation and delivery of dsRNA for anti-viral feed supplementation.

One key factor is the scalable production of genetically engineered *C. reinhardtii* strains. In this regard, it was previously reported that such transgenic strains, generated through the same chloroplast transformation process [193,215], can be readily scaled in photo-bioreactors to 100 L [218]. This provides evidence at the pre-pilot scale that such microalgal-mediated dsRNA production could be implemented in larger-scale reactors. Another interesting issue is the state of the delivered algae for high protection efficiency and cost-effectiveness. It is important to consider the survival of living transgenic microalgae in the environment, so using dried or inactivated cells would be an ideal way of applying the genetically engineered strain. However, balancing with the additional processing costs, delivering the microalgae producing dsRNA as living cells either by accumulation in *Artemia*, for improving delivery efficiency, or using microalgae paste (wet cell pellet) would save the cost of the drying process. In addition, due to the fact that the transgenic microalga is free from any antibiotic resistance gene, the use of live cells containing only a fragment of the target gene(s) should be reconsidered for non-harmful use in farm applications.

### 5.2. Nano-Containers

In general, the definition of nanoparticles includes materials with at least one dimension and with a size between 1 and 100 nm [219]. Due to their biocompatibility, low toxicity, and biodegradability, nanoparticles are also a promising delivery platform for different applications, such as gene therapy [220], miniature bioreactors [221], vaccination [222], and disease diagnosis [223]. However, only a few types have been used for the administration of bioactive compounds in shrimp. Among these are virus-like particles (VLPs), DNA nano-containers, liposomes, and chitosan nanoparticles. This approach would be particularly attractive for the nanoencapsulation of purified dsRNA, but not for its use with whole bacterial cells. Furthermore, the increase in the cost related to nanoencapsulation can be justified due to the improvements in the lifetime and administration of dsRNA to target cells, in comparison with the whole bacterial cells approach; therefore, the quantities of dsRNA used as feed additives could be significantly lower [69]. Currently, the vast majority of nanocarriers have been tested only through injection; nevertheless, oral delivery experiments are in progress [224].

### 5.3. Nanoparticles of Chitosan

Chitosan, a cationic polymer derivate of chitin, is the principal biopolymer of the crustacean exoskeleton, including those of shrimp and crabs. Chitosan can be used for the encapsulation and administration of nucleic acids, both in vitro and in vivo [225]. Interestingly, Rajeshkumar et al. [226] reported that after one week of feed administration of a chitosan-attached DNA construct targeting the VP28 capsid protein of WSSV, there was a relatively long-term protective effect. According to those authors, the challenge with WSSV at one, two, and three weeks after stopping the delivery of dsRNA produced survival rates of 85%, 65%, and 50%, respectively [226]. Importantly, even the result at three weeks after administration may be feasible at the field scale, as a survival rate of 50% is still profitable [165].

### 5.4. Liposomes

Cationic liposomes are defined as spherical lipid vesicles that have positively charged functional structures on both sides. Owing to electrostatic interactions, the cationic liposomes can more efficiently interact and encapsulate anionic molecules, such as peptides, acidic proteins, and nucleic acids, in comparison to neutral or anionic liposomes [220]. Moreover, cationic liposomes can also enhance cellular uptake due to the positive charges on their surface and via their attraction to the negatively charged exterior surface of the cell [227].

For shrimp-linked applications, cationic liposomes have been tested for the delivery of dsRNA [228,229], whereas their anionic counterparts have been used for delivery of recombinant VP28 protein against WSSV [230]. Apiratikul et al. [228] described how the delivery of dsRNA by injection, targeting the protease gene of YHV, and encapsulated in cationic liposomes, improved protection against YHV. The administration of liposomes containing dsRNA-YHV showed a 10% cumulative mortality for two weeks, compared to 50% mortality in the case of non-encapsulated dsRNA-YHV [228]. Furthermore, the use of cationic liposomes to encapsulate dsRNA not only decreases the effective quantity of dsRNA needed for viral inhibition but can also increase the lifetime of the dsRNA when it is delivered through injection. For example, in the case of cholesterol-based cationic liposomes used to encapsulate dsRNA-YHV, just 0.05 μg of dsRNA-YHV/g shrimp was sufficient to accomplish the same degree of YHV inhibition reached with 1.25 μg of non-encapsulated dsRNA-YHV/g shrimp. Moreover, at the same level of dsRNA concentration, the liposome-based system showed 50% mortality (sixty days after dsRNA delivery), whereas the group of animals injected with non-encapsulated dsRNA reached a mortality of 90% [229].

Additionally, the liposome-mediated approach can also be used for oral administration, as the liposomes can protect the encapsulated dsRNA from shrimp digestive enzymes, thus increasing the dsRNA response [229,231,232]. In comparison to the administration of *E. coli* expressing dsRNA-Rab7 implanted in agar [200], the liposome-based strategy could reduce the quantity of dsRNA needed for oral administration by ten-fold [229]. However, the quantity of dsRNA needed to prevent the disease caused by YHV is still around 400 times more than the effective dose achieved through injection [166]. Therefore, more improvements are needed, not only in the structure of liposomes, but also in the generation of liposome-supplemented feed to improve the absorption efficiency. These methods all have the goals of raising dsRNA concentration levels to those reached by injection and making the deployment of liposome-based approaches economically viable [69].

The use of lipid-mediated nanocarriers, such as lipoprotein particles and vesicles, is widely recognized as an intercellular transmission strategy for the relocation of small molecules, such as miRNA and siRNA, between cells. These nanocarriers may also offer protection from ribonuclease activity [231,233]. For example, He et al. [234] reported a reconstituted high-density lipoprotein structure, termed a nano-lipoprotein particle. These nanoparticles have a positive charge when mixed with cationic lipids, which allow them not only to interact with RNA structures, but also generate protection against RNase digestive activity. Another potential strategy is related to the use of exosome-mimetic nano-vesicles. These vesicles have a characteristic lipid bilayer membrane that can potentially include diverse molecules, such as proteins, lipids, and nucleic acids, that can be delivered and taken up by cells [235]. Given the potential of these vesicles to transport bioactive compounds to cells, siRNA packaged into exosomes mimics nano-vesicles and can be successfully absorb by cells. Nevertheless, such approach still faces limitations, mostly related to the low yield obtained from cell cultures [236].

### 5.5. Glucan Particles

Interestingly, Zhu and Zhang [237] reported that in *M. japonicus*, the administration of VP28-siRNA encapsulated into β-1,3-D-glucan particles led to protection against WSSV. Moreover, the data suggested that β-1,3-D-glucan-encapsulated VP28-siRNA particles (GeRPs) might be released in the shrimp hemocytes and produce a higher inhibition level of WSSV replication, compared to naked VP28-siRNA. Therefore, the delivery of GeRPs harboring VP28-siRNA significantly reduced the mortality rate of shrimp infected with WSSV and constitutes a new potential prophylactic and/or therapeutic approach to manage shrimp pathogens [237].

### 5.6. Virus-like Particles (VLPs)

VLPs are defined as nanoparticles that are shaped by the repetitive array of specific viral capsid proteins. The VLP icosahedral structure and disposition of particular capsid protein domains that are responsible for nucleic acid encapsulation inside and the host interaction outside have promoted their use as nanocarriers for the administration of nucleic acids to target cells [238].

VLPs are viral capsids that maintain the conformation and host-binding activity of native viruses but do not have the genetic material and, thus, are not infectious. VLPs are generally synthesized by self-assembly of viral capsid proteins produced by heterologous expression in a cell platform, such as *E. coli*, and can be used as bio-nanocarriers. VLPs have been synthesized from more than thirty different types of viruses for the administration of bioactive molecules and compounds, such as drugs, peptides, proteins, and nucleic acids. Moreover, VLPs are protease resistant, which makes them promising candidates for oral administration [239,240]. Nowadays, several VLP-mediated vaccines for humans and animals have been approved for commercial use, whereas several more are in different stages of clinical trials [239,241]. In the case of shrimp aquaculture, VLPs have been generated from capsid proteins of non-enveloped viruses, such as *Penaeus stylirostris* Densovirus (*Pst*DNV) [242], formerly called IHHNV [243,244], *Mr*NV [245], and *Pm*DNV [246].

The IHHNV-VLPs were the first characterized VLPs derived from a shrimp virus [243]. Based on a recombinant capsid protein expressed in *E. coli*, the crystal structure shows that the VLP is made up of sixty copies of the capsid protein protomers, which makes it the smallest parvovirus virion reported to date [247]. As in the case of the VLPs obtained from other viruses [245], the IHHNV-VLPs have been reported to randomly incorporate non-specific nucleic acids, mainly RNA. Moreover, IHHNV-VLPs can enter the shrimp’s primary hemocytes [243]. Recently, it was reported that simultaneous VP37 and VP28 dsRNA encapsulation in IHHNV-VLPs improves shrimp protection against WSSV. The researchers demonstrated that co-encapsulation of dual dsRNA had a better WSSV-silencing effect compared to the naked dsRNA. Furthermore, they showed that co-encapsulation of dsRNA into IHHNV-VLP increased not only the count of hemocytes but also the phenoloxidase activity needed to fight against an acute WSSV infection. Thus, this synergetic effect acts to delay shrimp death and to lower the cumulative mortality rate, compared to both non-encapsulated dsRNA and control groups. Such results shed light on a possible new approach to manage viral infections in shrimp aquaculture [248].

*Mr*NV virions have an icosahedral structure with a diameter of 27 nm and are derived from a specific capsid protein. The first *Mr*NV-VLPs were reported by Goh et al. [245], and the implementation of *Mr*NV-VLPs as nanocarriers for aquaculture usage was first described by Jariyapong et al. [249] using the recombinant capsid protein of *Mr*NV, which was generated in *E. coli*. The *Mr*NV-VLPs have several biochemical characteristics that make them a potential alternative for the bio-encapsulation of dsRNA and for its application in shrimp aquaculture [250]. For example, Citarasu et al. [251] showed that, in the case of *M. rosenbergii*, oral delivery of baculovirus-expressed *Mr*NV-VLPs generated a protective immunity response against *Mr*NV infection, which translated to a survival rate of 78%.

Recently, the in vitro assembly of *Pm*DNV-VLPs produced in *E. coli* was also reported [246]. According to these authors, the *Pm*DNV-VLPs may be a promising tool for the administration of nucleic acids to stimulate the shrimp’s innate immune response. More recently, Sinnuengnong et al. [242] revealed that delivery of co-expressed *Pst*DNV-VLPs and dsRNA-YHV-Pro (targeting YHV protease), produced in the same bacterial cells, also conferred protection against YHV infection, represented by a higher degree of YHV suppression and lower shrimp mortality compared to the administration of un-encapsulated dsRNA-YHV-Pro. Thus, *Pst*DNV-VLPs are a feasible bio-nanocarrier for dsRNA administration, which can maintain the shrimp anti-viral activity of the dsRNA for a longer time, in comparison to naked dsRNAs [242].

Interestingly, the Ca^2+^ ion-dependent oligomerization of the capsid protein monomers to form VLPs represents an opportunity to lower the amount of randomly incorporated nucleic acid in the VLPs. After deconstructing the VLPs through Ca^2+^ reduction, the un-specifically encapsulated nucleic acid can be eliminated by protein digestion before the reconstruction of the VLPs in the presence of a specific dsRNA of choice, plus Ca^2+^ ions [69,249]. Moreover, a sensitive and simple methodology to check the disassembly and reassembly of VLPs by using the protein fluorescence spectra (300–440 nm) is currently available [252]. For example, purified VLPs of human papillomavirus (HPV-16 L1) revealed a significantly higher fluorescence signal, in comparison to VLPs disassembled by the action of a reducing agent, in which the fluorescence was almost completely quenched. Therefore, by depletion of the reducing agent, the fluorescence is restored to its basal intensity, signaling the reassembly of the VLPs. Such an approach might be used in the case of VLPs derived from other viruses [252].

Through injection, VLPs delivered twice as much dsRNA-VP28 (24 h post-infection (hpi)), in comparison to non-encapsulated dsRNA. Nevertheless, at 72 hpi, the quantity of dsRNA administered through both strategies was almost the same [253]. In terms of silencing efficiency, both administration methods showed VP28 silencing at 48 hpi. However, the knock-down efficiency of the dsRNA-VP28-VLP was significantly higher in comparison to non-encapsulated dsRNA-VP28. It was suggested that the increased initial taken-up rate could be due to the possibility that the VLPs were administered through two different routes: (i) a receptor-based route mediated by host cell identification of the capsid C-terminus protomer (hypothetical protruding domain), or (ii) by a micropinocytosis system [253]. Moreover, the dsRNA-VP28-VLPs were shown to produce higher protection against WSSV, in comparison to non-encapsulated dsRNA-VP28. The mortality rate of the animals injected with dsRNA-VLPs was 30%, whereas the group injected with non-encapsulated dsRNA reached 45%. Higher protection from the VLP-mediated administration was suggested to be caused by either an increased efficiency of administration of the dsRNA or by a mixed effect caused by the RNAi and the immuno-improvement originated by the presence of the VLPs [253]. The latter point is based on the fact that shrimp pre-inoculated only with empty *Mr*NV-VLP also induced a lower viral load, in comparison to the positive control. In addition, RT-qPCR assays showed that key genes related to the viral immune response [254] were significantly overexpressed in the case of the dsRNA-VP28-VLP treatment, in comparison to the non-encapsulated dsRNA-VP28 treatment group.

More recently, Wuthisathid et al. [255] described the dual-expression of dsRNA and a viral capsid protein in *E. coli* strain DualX-B15 (DE3). This novel *E. coli* strain, which can express recombinant proteins simultaneously (such as capsid proteins to produce VLPs) with dsRNA, was genetically modified to be both protease- and RNase-III-deficient through P1 phage transduction. This cost-effective platform could be very useful in the context of shrimp anti-viral therapeutics’ production [255].

VLPs could be a potential platform that can be integrated into current administration approaches, such as *Artemia* bio-encapsulation and coating of feed pellets, similar to that employed in the case of the cationic liposomes [229]. VLPs might not only potentially reduce the working concentration of dsRNA, but also improve the shelf life of dsRNA by protecting the dsRNA against nuclease activity [69]. Several biological and chemical engineering methods have been developed for VLP alterations. Chemical modifications can be implemented using reactive compounds or molecules linked to the amino acid side chains on the outer and inner surfaces of the VLPs to promote dsRNA encapsulation and/or tissue tropism [256]. For instance, site-directed mutagenesis has been employed to expand the positive charge net of the VLP interior to engage the dsRNA molecules. Moreover, the consistent development of ligands accomplished through an exponential enrichment (SELEX) method might be used to recognize aptamers for adherence to either side of the VLPs. Those modifications can be used to optimize not only the cargo-loading process but also the tissue tropism [257,258,259,260].

## 6. RNAi Inhibition

Viral evolution has led to the development of different mechanisms for evasion of the host RNAi systems, making the infected cells more acquiescent to viral infection. For example, inside host cells, the *Mr*NV uses the protein B as a strategy to achieve the inhibition of viral RNA elimination in a sequence-specific way. Generally, the B2 protein of Nodaviruses has the capacity to interact with dsRNA (>10 bp), therefore, preventing siRNA production via Dicer activity [261]. Furthermore, the silencing of this protein by sequence-specific dsRNA showed a reduction in the mortality of red-claw crayfish (*Cherax quadricarinatus*) [261] and *M. rosenbergii* [201] infected with *Mr*NV. *Mr*NV is the etiological agent of white tail disease in giant freshwater prawn, which generates a significant economic impact on the aquaculture sector. In the case of shrimp, there is no well-characterized viral suppressor mechanism of the RNAi system; however, there is some information that points to WSSV inhibiting the RNAi response [102] and some hypotheses that suggest that IMNV encodes a protein with a dsRNA-binding domain that might function as a suppressor of the RNAi mechanism [8,262,263].

## 7. General Limitations of RNAi Technology

RNAi technology has become a key tool for the functional manipulation of gene expression and is currently considered as one of the most promising approaches to manage viral diseases in the shrimp aquaculture sector. Nevertheless, despite the initial enthusiasm about this outstanding system for sequence-specific gene regulation, there are several challenges that must be met before the RNAi technology can be deployed for practical applications in the aquaculture industry. These include the effective delivery of dsRNA in vivo and the minimization of off-target effects. This is important, as the knock-down of un-targeted genes that have partial sequence identity to dsRNAs/siRNAs/shRNAs has been reported [41,264].

The off-target activity is triggered by a partial sequence homology that leads shRNA/siRNA to eliminate the mRNA of non-intended genes. This situation can not only potentially result in undesired toxicity but also adds complexity to the explanation of phenotypic effects [265], and the design of optimal shRNA/siRNA effector molecules is one of the principal approaches deployed to reduce off-target effects. Previous reports have stated that different variables can affect the specificity of shRNA/siRNAs, including the thermodynamic properties [266], GC content, starting nucleotide [267], selected target region [268], size [34], and the presence of palindromes [269]. Therefore, diverse tools for computational design have been used to systematically and accurately determine, on a transcriptome-wide scale, the potential unintended RNAi effects among siRNA and target sequences [270,271,272].

Regarding the risk assessment analyses of potential off-target gene knock-down, the European Food Safety Authority (EFSA’s GMO Panel) considers that a set of in silico variables allows the identification of unintended targets. Bioinformatics analyses for off-targets are based on several criteria that predict the silencing efficiency [273]. Thus, in silico target identification algorithms are created mainly based on a criterion linked to the biochemical and thermodynamic features of sequence complementarity, among other variables [274]. Based on the above, a bioinformatics-mediated approach for the risk assessment related to endogenous RNAi unintended targets could be applied according to the guidelines developed by the EFSA’s GMO Panel. Moreover, for the identification of possible effects on non-target organisms (NTOs) in the environment, bioinformatics analyses may detect which NTOs have genes that present some degree of sequence similarity compared to the target gene [274]. These data may be employed to identify the NTO that requires special consideration throughout the risk assessment process. Importantly, no additional risk assessment may be necessary if the lack of minimum sequence similarity is reliably confirmed for a RNAi response [275,276].

Since shRNA/siRNA depends on the endogenous miRNA system to accomplish a strong target knock-down, the presence of higher levels of exogenous RNAs can result in saturation of one or more elements of the endogenous RNAi cascades, leading to potential toxicity and mortalities in the treated organisms [277]. Based on this fact, the delivery of the siRNA to the minimal possible concentration is strongly recommended, as RNAi is generally highly efficient at minimal levels [278]. Thus, the employment of lower doses of shRNA/siRNA is an approach generally used to improve the knock-down specificity through significantly lowering the likelihood of unintended effects (specific and unspecific) [278,279,280,281,282].

Apart from unintended effects (off-target), the administration of an RNAi effector to particular cells or tissues remains as one of the key limitations for the deployment of effective and safe RNAi therapies in vivo, as siRNAs/shRNAs have not only a low cellular absorption, but also a non-specific tissue distribution, low stability, and rapid excretion [283]. Thus, some chemical changes, such as alterations in the RNA backbone, the substitution of specific nucleotides with analogues, and the adjunction of conjugates, could be incorporated into the RNA effectors to improve the half-life, both in intracellular and extracellular conditions [284]. For example, because of its size and negative charge, naked or un-encapsulated siRNA is generally incapable of crossing the cellular membrane to penetrate the target cell cytoplasm, which is a critical factor for an effective knock-down effect [285]. So, suitable administration systems are needed to enhance not only the cellular accumulation of RNAi effector molecules, but also to improve the translocation from endosomes to cytosol [286]. Several administration approaches, including viral vectors, aptamers, cholesterol, antibodies, calcium phosphate, cationic lipids, and nanoparticles [287], have been extensively tested for their accessibility to diverse tissue types by different administration routes. Nonetheless, the wide diversity of variables and requisites makes the development of a “universal” in vivo administration method, appropriate for every potential scenario of RNAi usage, unlikely [5].

Even if some of the issues related to the administration, efficiency, safety, and cost–benefit analysis of RNAi applications can be elucidated, the questions about consumer acceptance remain, as the shrimp will be used for direct human consumption. Generally, the key problem from the consumers’ perspective is linked to safety issues. In this regard, the use of isolated dsRNA may not represent safety issues, in comparison with some other possible administration methods. For instance, the use of bacterial platforms, incorporating vectors with chromosomal elements and/or antibiotic resistance genes, would not be considered safe in some countries, even if the bacteria were inactivated [69].

### RNAi Limitations in the Aquaculture Sector

Selecting a suitable administration methodology for each aquaculture farming scenario is one of the key challenges to making RNAi a practical and realistic tool at an industrial level. Administering RNAi therapies to inland systems has some benefits over open-environment aquaculture systems, not only with respect to the waste-control process, but also the direct availability of animals and laboratory installations. In the case of shrimp growth installations, the large number of individuals raised in ponds/tanks makes injection approaches impractical for commercial applications at the field level. On the other hand, the immersion of organisms in a solution with RNAi effector molecules synthesized in vitro is a non-invasive and effective approach extensively applied in shrimp RNAi experiments. However, such an approach can also become increasingly costly and time-consuming, as large quantities are generally needed to accomplish a continuous target gene knock-down. Moreover, in the case of shrimp, it has been reported that synthetic dsRNA can shorten the RNAi effective time because of the limited half-life when delivered in vivo [84]. Thus, for in vivo applications, more studies are needed to not only create high-expression plasmids, but also to improve the transfection strategies used to produce RNAi effector molecules. Currently, the administration of these effector constructs through oral delivery is considered the most viable approach for land-based aquaculture applications [288].

On the other hand, another factor that hampers not only the understanding of the shrimp RNAi mechanism, but also the pathogen–host interplay at the molecular level, is the lack of a stable cell line, which is a key issue for the development of methods for control and management of pathogens [288]. A promising strategy that may be applied to overcome some of the administration problems linked with the development of RNAi application at the industrial level could be the generation and use of transgenic organisms (bacteria or microalgae) that function as platforms to express RNAi effector molecules to knock-down key conserved genes of specific pathogens [23]. Nonetheless, deploying such genetically modified organisms (GMO) to feed shrimps will imply additional safety and environmental-related concerns.

Since the last decade, the studies on dsRNA-based viral management in shrimp aquaculture have shifted from the impractical and tedious delivery of naked dsRNA via injection to a more practical oral delivery approach. So far, although those studies showed good results, the vast majority have been implemented in the laboratory, scarcely ever at the pilot scale, and never at the farm level. Additionally, there have not been attempts to standardize the methodological strategy used in the different studies; thus, the comparison between the efficiency of the diverse tests can be difficult and sometimes even impossible. So, a strategy to test the efficiency of the different administration methods should be standardized to control parameters, such as the choice of dsRNA, viral load, stocking density, source of shrimp, and the viral challenge model. The establishment of such methodologies will also be helpful in gaining knowledge about the potential immunostimulatory impact of the administration strategies proposed by different researchers [69].

Most importantly, the feasible application of RNAi technology at an industrial level will be dependent on lowering the associated costs. Currently, an estimation for the supplementation of shrimp feed with dsRNA targeting WSSV is that it would increase the cost of the feed by at least 10-fold. This estimation was extrapolated from the cost of the synthesis of dsRNA through a fed-batch system using a 10 L fermenter [182], which is the largest-scale dsRNA-generation method applied to shrimp aquaculture described to date. Such a price tag is a strong limitation for RNAi applications at the field level. Regarding this point, in the case of bacterial expressed (*E. coli* HT115) dsRNA, the estimated synthesis price would be EUR 43.2/mg, whereas ordering through commercial companies would be ≥USD 50/mg, and the production by commercial kits (such as the MEGAscript^TM^ RNAi Kit; ThermoScientific, Vilnius, Lithuania) would be around USD 300/mg. It is important to highlight that these prices can change over the time and depend on the country [190].

Additionally, feed containing dsRNA has been shown to be effective in a therapeutic mode, so it could be used exclusively after a viral pathogen is detected; therefore, the farmers might calculate the potential convenience of the supplementary feed cost during a narrow timeframe. In the case of the prophylactic mode using intermittent or continuous feeding, not only is there a requirement for an additional reduction in dsRNA synthesis costs, but also an enhanced stability and efficiency would be needed to make the RNAi approach more appealing to shrimp producers [69].

Therefore, the potential deployment of the RNAi technology applied to pathogen control in shrimp aquaculture depends not only on the particular goal, but also on the strategy deployed, timeframe, and point of application. For instance, the potential use of injection strategies is more related to broodstock in hatcheries and/or shrimp-breeding centers, whereas in the case of juveniles, PL and larvae grow in ponds, and the one feasible strategy is oral administration. Considering this perspective, successful oral administration will be first applied in hatcheries, where the higher shrimp density and lower culture volume will allow a more efficient administration. Hopefully, in the near future, further cost reductions will permit grow-out pond applications [69].

RNAi-based methods have proven to be effective against viral pathogens. Nevertheless, cellular stability and delivery are two key challenges that must be overcome to develop RNAi-based therapies in the future [65,68,94,97,145,153]. Moreover, the efficacy of RNAi-based therapies depends on several key factors, including the choice of the target pathogen [142,143], gene [68,94,99,144,154], tissue [151,152], and the administration route [146].

This scenario suggests that many challenges must be overcome before the RNAi technology can be solidly established as a safe and effective strategy for the control of pathogens of economically important species. Thus, before this technology can find a practical application in aquaculture, some hurdles need to be addressed, especially in relation to the enhanced stability of the RNAi molecules and accomplishing particular and controllable long-term knock-down effects. Moreover, the development of cost-effective and efficient administration strategies without compromising the environment is another of the key challenges for the deployment of RNAi technology at the industrial level [5].

Thus, before the RNAi-mediated therapies find practical and realistic usages for the control of viral pathogens in aquaculture, several hurdles must be resolved, such as its cost, influence on the environment, and a deeper characterization and knowledge about the RNAi mechanism at the molecular scale. In reality, it is costly and complicated to design dsRNA administration programs for protection from pathogens, since they are mostly unpredictable. Therapeutic aspects, though exhibiting significantly lower efficiency than prophylaxis, are still needed by farmers to help them gain an adequate harvesting time before 100% mortality. This will generate a new era of aquaculture and support several Sustainable Development Goals (SDGs) proposed by the United Nations, which aim to sustain all living organisms and enhance food (seafood in this case) security. However, despite these challenges, RNAi technology represents a very promising tool for the development of new therapeutic and/or prophylactic strategies deployed for the management of viral pathogens that affect the aquaculture industry [41].

## 8. Safe Use of RNAi

The deployment of RNAi on an industrial level has also been investigated for its implications of some safety issues. In this regard, it has been reported that dsRNAs were not detected in *M. rosenbergii* tissues, including hemolymph, after 7 days, and the expression of the target knock-down gene was fully recovered after 28 days. Importantly, the effect of RNAi therapy was not inherited to the offspring [289], and did not interfere with the fecundity and reproduction of the offspring generations (three generations were tested in this study) [42]. For example, female shrimp injected with a specific anti-viral dsRNA generated an offspring without any detectable quantities of the specific dsRNA used in the therapy; nonetheless, the administered dsRNA was detected in diverse organs, including the ovaries. However, the specific dsRNA was not detected in any of the larval (protozoea, mysis, nauplii, and eggs) or post-larval stages [290].

Additional studies should be carried out to investigate if the RNAi effector molecules could generate genetic alterations related to mechanisms of regulation of expression mediated by epigenetics changes. So far, there is no evidence that points to any negative effects linked to the use of the RNAi technology in the aquaculture sector. Moreover, the deployment of this technology has a low likelihood of environmental issues that might be generated by a possible escape of genetically modified populations. For example, in the case of the prawn *M. rosenbergii*, a recent study showed that dsRNA-mediated manipulation manifests via a temporal and post-transcriptional route (siRNA pathway). The temporal effect of this route further supports the safety of employing this RNAi-mediated technology in environmental and aquaculture usages [42,289]. There are not many reports regarding the general health status of the cells subjected to RNAi-based gene knock-down; nonetheless, the available information indicates that cells not subjected to RNAi are healthier than their treated counterparts. Indeed, there must be a metabolic burden related to the process of RNAi; thus, this could presumably translate into a lower biomass production [45].

On the other hand, genome manipulation and/or genetic modification applied to food products are generally not accepted by consumers in some geographical regions, mainly due to the potential risks and long-term unintended effects, as well as misinformation [291]. Importantly, the implementation of the RNAi technology only generates temporal changes in the animals treated; therefore, the animals produced are considered as non-GMO [292].

Despite the current limitations, RNAi is a powerful genetic tool that causes minimal non-intended perturbations. Many efforts have been concentrated on the generation of an efficient and safe technology that could be used not only to manipulate gene expression without any extensive and permanent invasion but also might be adapted for a low cost in large-scale in vivo applications in the aquaculture sector. Moreover, as a reverse genetic tool, the deployment of RNAi is a very promising strategy for the development of the biological drugs sector, both in animals and humans [45].

Nowadays, in the aquaculture sector, the commercial application of RNAi technologies has been especially hindered by issues related to delivery and cost. However, some successful applications have been developed to solve some production-related issues, such as female shrimp spawn stimulation [293], and the generation of all-male prawns [292]. Currently, this last case is the main example of the commercial-scale use of RNAi applied to the shrimp aquaculture sector [224].

## 9. Other Applications of RNAi-Mediated Gene Silencing to Crustacean Aquaculture: Mono-Sex Culture and Alternatives to Avoid Eyestalk Ablation

For various aquaculture species, the process of mono-sex generation is highly advantageous and desirable for the industry, as some sex-dependent growth differences can be obtained by such a process [294,295,296]. For example, in the case of freshwater prawn (*M. rosenbergii*) [297] and Nile tilapia (*Oreochromis niloticus*) [294], male individuals can grow quicker and attain increased harvest weights compared to females. Additionally, when sex-reversed males mated with normal males, they showed a regular reproductive behavior but generated only male offspring of homogametic sex [298]. Such culturing of all-male prawns (ZZ) was successfully developed over three generations without producing any detrimental side effects [42].

**Figure 4:**
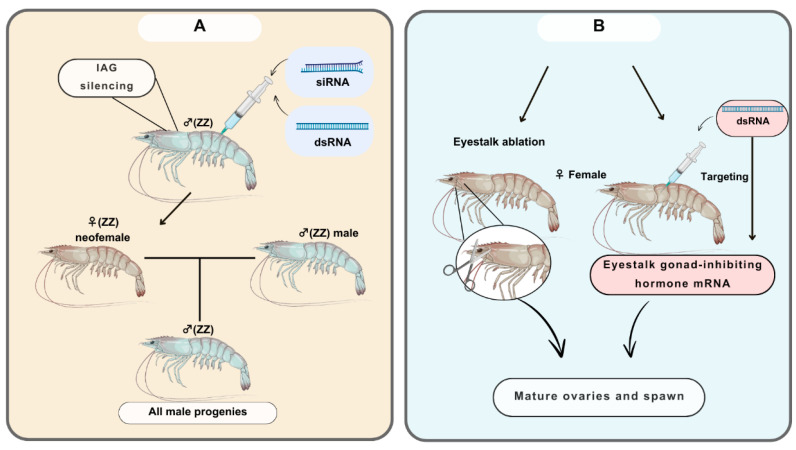
RNAi technology applied to shrimp reproductive issues. (**A**) Generation of all-male offspring. (**B**) Alternative method to eyestalk ablation.

Following the characterization of the insulin-like AG hormone (IAG) in *M. rosenbergii*, RNAi was employed to silence the expression of the sexual-differentiation-mediated IAG gene [298]. When the silencing was carried out at an early developmental phase of juvenile males, identified by using molecular sex markers [299], the IAG knock-down led to a complete and functional sex transformation of males into neo-females. Moreover, these neo-female individuals could successfully mate with regular males, generating an all-male progeny [300]. The development of this process established a milestone, being the first commercially available approach that does not require the use of hormones/chemicals or any genetic modification of the treated organisms to successfully accomplish its objective of generating an all-male progeny (Figure 4A). Importantly, the RNAi-mediated effect occurs through the post-transcriptional route, and the temporal effect of this route further supports the safety of this RNAi-mediated technology in aquaculture [42]. Furthermore, since the manipulation is temporal and performed on parents, its effects are not transmissible to subsequent generations; therefore, such therapy is exempt from the regulatory obstacles inherent to genetically modified crops [301]. In the coming years, based on these advantages and with further enhancements in the administration processes, we will likely see more commercial applications of gene knock-down related to several aspects of shrimp culture, and aquaculture in general [28].

In another example, RNAi technology was implemented to supply an alternative methodology for eyestalk ablation in cultured *P. vannamei* (Figure 4B). In this case, the RNAi method was deployed to provoke female shrimp to develop mature ovaries and spawn. The delivery, by injection, of dsRNA targeting the inhibition of the gonad hormone of *P. vannamei* females showed a reduction of 64% in the levels of expression of the eyestalk gonad hormone [293]. In this case, the implementation of the RNAi technology permitted a less invasive manipulation that avoided the traumatic and stressful process of removal of shrimp body parts.

## 10. Conclusions

RNAi technology has been established as a powerful tool not only for the study of functional genomics but also as a very promising technique for the development of therapies for the management of various viral and bacterial pathogens that seriously affect the crustacean aquaculture sector. Currently, the main approaches focus on the search and refinement of oral administration methods, mainly using expression platforms, such as bacteria (*E. coli*) or microalgae (*C. reinhardtii*). These methods, in conjunction with other approaches, such as the use of enriched *Artemia* and/or the incorporation of VLPs, could mean a substantial advancement in the implementation of such systems. In addition to the different factors that affect the design and effectiveness of the different approaches tested so far, which are based on the objectives proposed in each case, one of the main challenges that must be overcome for large-scale deployment in the aquaculture sector relates to economic viability. However, recent advances, such as the establishment of safe therapies to obtain mono-sexual populations, open the possibility for the development of various commercial applications of RNAi technology in the crustacean aquaculture sector.

## Figures and Tables

**Table 1:** Key elements of the RNAi machinery.

Effector Molecule	Structure	Key Interacting Proteins	Type of Silencing Effect
dsRNA (long)	>100 bp	Dicer, Argonaute, TRBP	RISC (temporal)
siRNA	21–22 bp seed (2nd–7th nt) and supplementary (12th–17th nt) regions	Dicer 2, Argonaute 2, TRBP1	RISC (temporal)
miRNA	21–24 bp seed (2nd–7th nt) and supplementary (9th–18th nt) regions	Dicer 2, Argonaute 2, Drosha, Dicer 1, Argonaute 1	RISC (temporal) RITS (permanent)
